# Corn variety identification based on improved EfficientNet lightweight neural network

**DOI:** 10.3389/fpls.2025.1603073

**Published:** 2025-06-19

**Authors:** Jinpu Xu, Jinhao Lan, Guangjie Lv, Dexin Ma

**Affiliations:** ^1^ College of Animation and Communication, Qingdao Agricultural University, Qingdao, China; ^2^ Shandong Smart Agriculture Research Institute, Qingdao, China; ^3^ College of Agronomy, Qingdao Agricultural University, Qingdao, China; ^4^ Network Information Office, Qingdao Agricultural University, Qingdao, China

**Keywords:** corn ear, variety identification, classification, EfficientNetB0, CBAM, dilated convolution

## Abstract

The authenticity of corn seeds is critical to yields and their market value. The screening of corn ears is an important step in the processing of corn seeds. In order to protect the intellectual property rights of corn varieties and realize intelligent ear screening, this article proposes an improved EfficientNet lightweight model, which uses deep learning technology to classify and identify corn ear images. First, 6529 RGB images of corn ears of five varieties were collected to construct a data set. Secondly, the number of MBConv modules in the EfficientNetB0 model was reduced, and the CBAM attention mechanism and dilation convolution were introduced to enhance the feature extraction capability. Finally, the Swish activation function was used to improve the stability of gradient transfer, and the SCD_EFTNet model was proposed. Experiments show that the proposed model has obvious advantages compared with mainstream models in indicators such as Recall, Precision, mAP, and inference time, and its mAP reaches 98.11%. The phenotypic characteristics of corn ears can be used to better classify and identify different varieties of corn, providing a reference for intelligent sorting of corn ears.

## 1 Introduction

As a widely planted crop in the world, corn is main food source and one of important industrial raw materials. Germplasm resources are an integral part of national food security and directly affect crop yield and quality. In developing countries such as China, Brazil, India, corn seeds IPR(Intellectual Property Rights) infringement, fake seeds and inferior seeds have occurred from time to time, causing heavy losses to breeding companies and farmers (Figueiredo et al., 2019; Auriol et al., 2023; Faria-Silva and Baião, 2023). Seeds are prone to confusion during planting, harvesting, transportation, storage and other production processes, and seed purity will affect genetic stability and its market value. Variety identification plays a crucial role in seed production, processing and marketing, which can protect the IPR of varieties and safeguard the interests of enterprises and farmers (Peschard and Randeria, 2020). On the other hand, the classification and screening of corn ears is an essential and important link in seed production and selection of new varieties. Traditional corn ear screening is labor-intensive, difficult to distinguish manually, and prone to errors. Therefore, there is an urgent need to develop a fast and accurate method for corn ear identification to improve the efficiency.

The identification of crop seed or disease by machine vision methods is mainly divided into two categories. One is to extract the features of the RGB images of seeds such as morphology, color, texture, etc., and then use machine learning methods such as BP neural network, k-nearest neighbor algorithm, and support vector machine to identify (Mallah et al., 2013; Koklu and Ozkan, 2020; Xiong et al., 2021). The other is spectral imaging technology, which extracts characteristic band information through near-infrared spectroscopy (Bai et al., 2020) or hyperspectral technology (Xu et al., 2023), and then combines PCA, partial least squares, etc. to identify the variety and authenticity of corn kernels.

In recent years, computer vision and deep learning technologies have developed rapidly and have been widely used in the identification of rice (Qiu et al., 2018), soybean (Zhu et al., 2020), pepper (Tu et al., 2018) and other crops, as well as in the process of grain purity, quality, and grade detection. In the field of plant protection, scholars have conducted multiple research projects on crop disease identification (Durmus et al., 2017; Deepalakshmi et al., 2021; Kaur et al., 2022). In the field of food safety, scholars have conducted research on geographical origin identification and traceability of agricultural products (Gao et al., 2019; Yan et al., 2020).

Compared with the complex process of feature extraction in machine learning, deep learning algorithms can automatically extract image features, and the extracted features are more effective and labor saving, so the recognition accuracy can be greatly improved. Convolutional Neural Network (CNN) is the representative of deep learning. It has the characteristics of self-learning, self-adaptation and strong generalization ability. In recent years, it has achieved satisfactory results in image classification, target detection, and face recognition (Traore et al., 2018; Veeramani et al., 2018; Nie et al., 2019). Most of the researchers achieved better performance and higher recognition rate by adjusting the parameters and network structure of the original CNN network. In recent years, precise detection technology of crop phenotype based on computer vision and deep learning has played an important role and has attracted widespread attention. Zhao (2021) et al. (Zhao et al., 2021) used the improved mobilenetV2 network to classify and identify soybean seeds with surface defects. The proposed sorting system can achieve high-precision and low-cost applications, with a total sorting accuracy of 98.87%. The picking speed is 222 seeds per minute. Tu (2021) et al. (Tu et al., 2021) adopted VGG16 and the transfer learning method, and proposed a non-destructive, high-efficiency, and low-cost identification method of single corn seed by scanning images of germ and non-germ surfaces of JINGKE 968. In order to detect fake corn seeds, Zhang et al. (2024) (Zhang et al., 2024) used hyperspectral and deep learning technologies to propose a deep one-class learning (OCL) network for seed fraud detection. The results show that the method has a mean accuracy of 93.70% for receiving real varieties and 94.28% for rejecting fake varieties, which is superior to several existing state-of-the-art OCL models. To achieve corn seed quality classification, Chen et al. (2022) (Chen et al., 2022) propose an improved ViT model SeedViT. The feasibility of SeedViT for classifying corn seed quality was studied and compared with DCNN and traditional machine learning algorithms, the result showed that SeedViT can be a new and novel way for maize seed manufacturing.

For the identification of corn varieties, previous research mainly focused on corn kernels. Although the identification of corn ears has not been common yet, Corn ears do contain rich genetic trait information, such as ear type aspect ratio, rows per ear, kernels per rows, kernels per ear,and kernel color, convex tip, axis section, etc (Warman et al., 2021; Zhou et al., 2021; Shi et al., 2022). These characteristics can be used to identify the authenticity of corn varieties. At the same time, although previous studies have confirmed the effectiveness of CNN in seed identification, they mainly implemented classification tasks based on large models and paid less attention to lightweight models. Large models have problems such as vanishing gradients, high computational costs, and large memory requirements (Tan and Le, 2019). The current trend is to implement lightweight architectures without affecting performance (Asante et al., 2024) (Heuillet et al., 2023).

Based on this, this study collected three-channel RGB images of five varieties of corn ears, improved its MBConv module based on EfficientNetB0, introduced the CBAM attention mechanism and dilated convolution technology, and replaced the ReLU function with the Swish activation function in the shared MLP, proposed a SCD_EFTNet lightweight network to identify corn varieties through ear phenotypic characteristics. The work of this article mainly includes data collection and preprocessing, simplification of the EfficientNetB0 model, training and parameter fine-tuning of the SCD_EFTNet model, ablation experiments on the strategies adopted in the model, and comparison with other mainstream models.

The remaining sections of this paper are structured as follows: The “Materials and Methods” section delineates the datasets and methodologies employed in this study. The “Results and Discussion” section systematically presents experimental findings, provides critical analysis of the outcomes, and examines potential limitations encountered during the investigation. Finally, the “Conclusion” section synthesizes key discoveries and their broader implications for the field.

## 2 Materials and methods

### 2.1 Experimental samples

**Figure 1:**
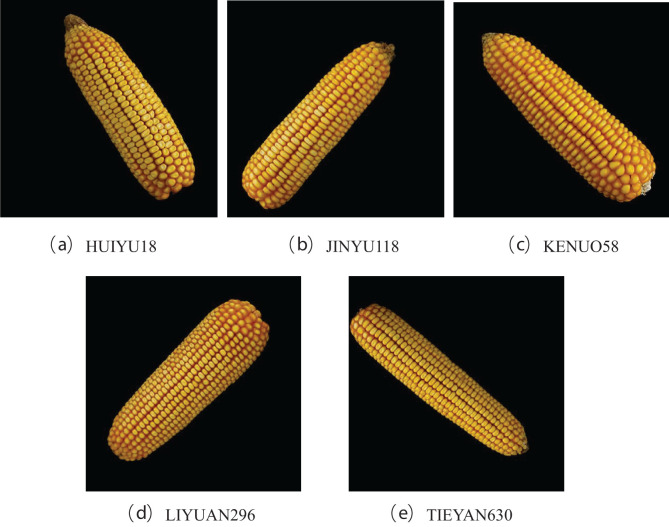
Images of corn ears from five varieties. **(a–e)** represent different varieties of corn.

In this paper, the experimental materials were selected from five different varieties of corn ears produced by Mizhou Seed Industry Co., Ltd. in Zhucheng City, Shandong Province, including KONUO 58, HUIYU 18, JINYU 118, LIYUAN 960, AND TIEYAN 630. Due to breeding needs, there is a certain genetic relationship between the five varieties, which makes it difficult to identify them manually. For each corn variety, the ears with intact phenotypes were harvested, and a Canon camera EOS 80D was fixed on a self-made stand for fixed-focus photography. The ears were placed horizontally on black light-absorbing flannel for photography, so that the background of the photo was black. Each corn ear rotates randomly around the cob and takes 3 pictures, resulting in a total of 6529 images, and the original images with a resolution of 3984 × 2656 pixels were obtained. The third library PIL (Python Image Library) of Python was used to convert the images into 500*500 pixels and the PNG format. The image examples of corn ears from different varieties are shown in **Figure 1**. All images were divided into training set, validation set, and test set according to the ratio of 7:2:1. The number of the images in the dataset is shown in **Table 1**.

**Table 1:** Numbers of the original image dataset.

Images for modeling	Training set	Validation set	Testing set	Total
HUIYU18	906	262	149	1317
JINYU118	939	268	152	1359
KENUO58	875	250	136	1261
LIYUAN296	885	253	144	1282
TIEYAN630	905	258	147	1310
Total	4510	1291	728	6529

Sufficient training set samples can avoid over-fitting and effectively improve the recognition rate and stability of the model, so data augmentation is often used to expand the data set (Shorten and Khoshgoftaar, 2019). In our experiment, we mainly adopt random rotation, scaling 0.2 times, horizontal flipping, increasing or decreasing exposure and other operations on the training set. The augmented images and the original image samples are used for training, which can further improve the robustness and adaptability of the model.

### 2.2 SCD_EFTNet model

**Figure 2:**
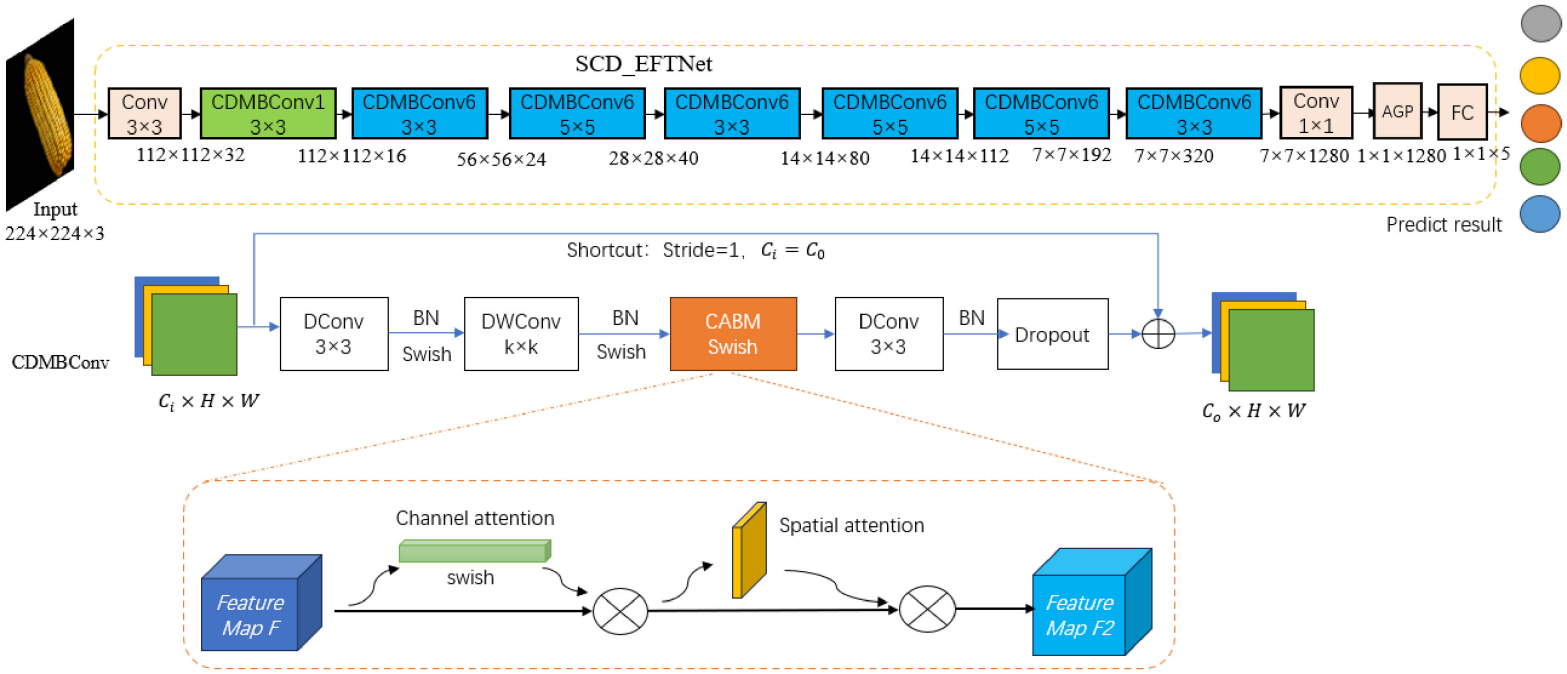
The structure diagram of SCD_EFTNet. Input data is 3 channels RGB images. 1/6 in CDMBConv1/6 represents the multiplication factor, which can expand number of channels of the input feature matrix. The last stage includes one common 1x1 convolution layer, average pooling layer (AGP), and full connection layer (FC).

Since corn ear images have similar characteristics in spatial feature distribution, color, shape contour, texture, etc., accurately classification can only be achieved by improving the fine-grainedness of the model. Based on the specific task of identifying corn varieties, this article considers that the EfficientNet network has many layers and is prone to overfitting. Therefore, the network structure needs to be improved, not only considering the accuracy and precision of recognition, but also considering the parameters size and storage space of the CNN model for embedded devices (Motamedi et al., 2022). Therefore, based on the EfficientNetB0 model, firstly, this paper reduces the number of layers and reduces the repeatedly stacked MBConv modules in the original network. Only one MBConv module is retained in each layer and a shallow network is designed; secondly, the MBConv module is improved, by introducing the CBAM attention mechanism, Swish function and dilated convolution, a lightweight SCD_EFTNet model for corn variety identification was designed. The network structure is shown in **Figure 2**.

#### 2.2.1 the feature extraction network

The task of this paper is fine-grained image recognition, which should extract sufficient semantic information from corn ear images. Therefore, the lightweight network EfficientNetB0, which is accurate, efficient and has a small model scale, is selected as the basic feature extraction network. The traditional deep learning model usually adjusts the network depth, width and resolution arbitrarily and independently, but the EfficientNetB0 model uses the composite coefficient *φ* to synchronize and coordinate the depth, width and resolution (Tan and Le, 2019). The formula is as follows in Equations 1, 2:


(1) $$d=\alpha^{\phi },\;w=\beta^{\phi },\;r=\gamma^{\phi }$$

(2) $$\text{constraits}:\{\begin{array}{c} \alpha \cdot \beta^{2}\cdot \gamma^{2}\approx 2\\ \alpha \geq 1,\;\beta \geq 1,\gamma \geq 1 \end{array}$$

**Figure 3:**
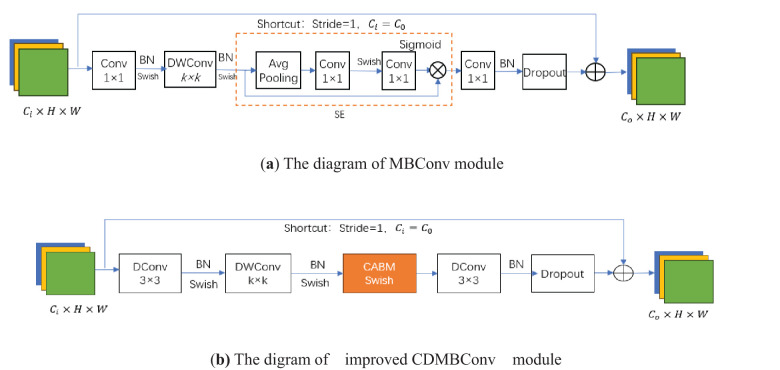
Comparison of the MBConv module with the improved CDMBConv module. **(a)** is the original module, **(b)** is the improved.

Among them, α, β, and γ are determined constants obtained by model search. The core module of EfficientNet-B0 is mobile inverted bottleneck convolution (MBConv). Firstly, This article reduces the number of repeatedly stacked MBConv, retaining only one MBConv in each layer of the backbone network;Then, the MBConv module is further improved by replacing two ordinary convolutions with dilation convolutions (DConv) to reduce parameter values and calculation amount. Then, the BN layer and Swish activation function are used to speed up the convergence speed of the model, and then DWConv(Depthwise separable convolution) is used to reduce the computational complexity of the model, which is also followed by the BN layer and Swish function. Next, the original SE module is replaced with the CBAM module to achieve channel attention and spatial attention. After passing through the BN layer, it is sent to Dropout, and finally added to the input feature map to obtain the output feature map. The above improved module is named CDMBConv. As shown in **Figure 3**.

#### 2.2.2 CBAM attention mechanism

**Figure 4:**
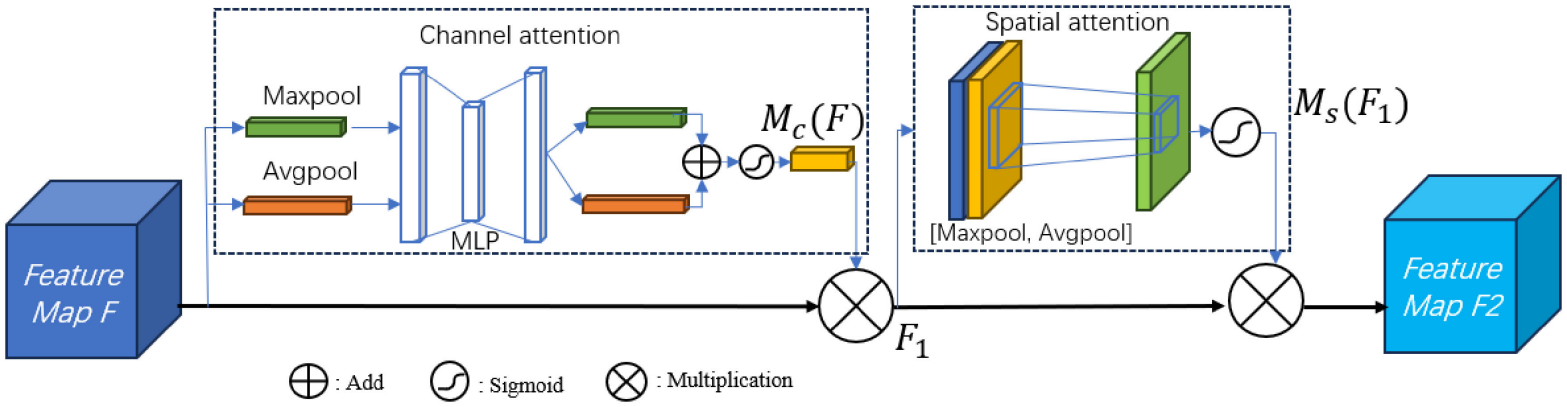
The process of CBAM.

The similarity in phenotypic characteristics of corn ears makes manual variety identification very difficult. The SE module in MBConv enhances the channel features of the input feature map by learning the importance of each channel. However, the SE module only learns channel features and ignores certain spatial pixel information in the image that is decisive for classification (Zhou et al., 2023), resulting in poor feature extraction results. This article embeds CBAM into MBConv, replaces the original SE module, performs average pooling and maximum pooling on the feature map F in the channel attention module, and then passes through 2 layers of shared MLP, and adds the output results through sigmoid function to obtain the weight coefficient Mc. Multiply the weight coefficient Mc with the original feature map F to obtain the new feature map F1. Next, after F1 passes average pooling and maximum pooling, the spatial attention channel splices it by channel, and the weight coefficient Ms is obtained after passing the sigmoid function. The new feature map F2 is obtained after multiplying the weight coefficient Ms and the feature map F1. The final feature map combines channel features and spatial features to enhance the image semantic information. The process of CBAM is shown in the **Figure 4** below.

The formulas are shown in Equations 3–5 below:


(3) $$\{\begin{array}{c} F_{1}=M_{c}\left(F\right)⊗F\\ F_{2}=M_{s}\left(F_{1}\right)⊗F_{1} \end{array}$$

The formula of channel attention is:


(4) $$\begin{array}{l} M_{c}\left(F\right)=\sigma \left(MLP\left(Avgpool\left(F\right)\right)+MLP\left(Maxpool\left(F\right)\right)\right)\\ \;=\sigma (W_{1}\left(W_{0}\left(F_{avg}^{c}\right))+W_{1}\left(W_{0}\left(F_{max}^{c}\right)\right)\right) \end{array}$$

The formula of spatial attention is:


(5) $$M_{s}\left(F\right)=\sigma \left(f^{7\times 7}\left[Avgpool\left(F\right),Maxpool\left(F\right)\right]\right)=\sigma \left(f^{7\times 7}\left[F_{avg}^{s},F_{max}^{s}\right]\right)$$

Among them, $W_{1}$ and $W_{0}$ is the weight of MLP, $F_{avg}^{c},F_{max}^{c}$ is the average pooling and maximum pooling features of channel attention respectively, $F_{avg}^{s},\;\;F_{max}^{s}$ is the average pooling and maximum pooling features of spatial attention respectively, and σ is sigmoid function.

#### 2.2.3 Dilation convolution

**Figure 5:**
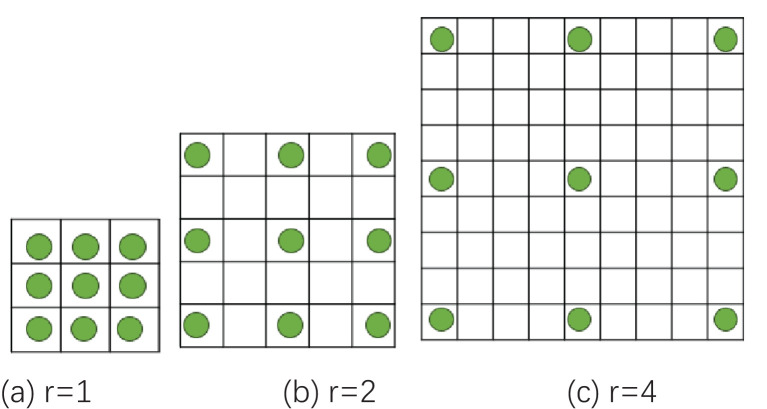
Schematic diagram of dilated convolution expansion. **(a)** r=1. **(b)** r=2. **(c)** r=4.

Dilated convolution introduces an expansion coefficient into traditional convolution, which expands the convolution kernel without increasing the number of parameters (Chen et al., 2017; Zhang et al., 2019). This allows the convolution kernel to extract more feature information, which is beneficial to model learning and classification. Corn ears are similar in phenotypic traits such as kernel color, row spacing, and contour shape, making it difficult to extract features of texture and contour edge details. The EfficientNetB0 network is formed by repeatedly stacking MBConv. Most channels of the ordinary convolutional layer are used to generate more detailed filters, thereby producing more complex parameters. At the same time, ordinary convolution is limited by the receptive field, has a single mode of capturing feature information on the image, and cannot effectively handle information of different scales and levels. This study replaces the two ordinary convolutions with dilated convolutions in the improved MBConv module, with expansion coefficients of 2 and 4 respectively, and also expands its convolution kernel to 3*3. **Figure 5** shows different expansion coefficients in dilated convolution.

#### 2.2.4 Swish function

The Swish activation function has better nonlinear properties than ReLU. The form of Swish function is shown in Equations 6, 7:


(6) Swish(x)=x·sigmoid(x)

(7) $$sigmoid(x)=\frac{1}{1+e^{-x}}$$

Compared with ReLU, Swish has non-zero derivatives across the entire real number domain, which helps to better propagate gradients during model training and avoid the vanishing gradient problem (Mercioni and Holban, 2020). The derivative of the Swish function is smoother near the zero point than ReLU, which helps to speed up the convergence of the model. In order to maintain consistency with the activation function in the MBConv module, this article replaces the ReLU function in CBAM with Swish, which is located after the first layer of neurons in the shared MLP. Some studies have shown that in some tasks, using the Swish activation function can lead to better performance (Jahan et al., 2023), such as higher accuracy or faster convergence.

### 2.3 Experiment environment

The processor used in our experiment is Intel(R) Xeon(R) Silver 4210 CPU @ 2.20GHz, 64GB memory, the GPU is GeForce RTX 2080, 11GB video memory, the CUDA version is 10.2, the operating system is Linux CentOS 7.6, and the model framework uses Pytorch 1.8.1, the programming tool is Jupyter notebook. In order to obtain better model performance, the experiment adopted a transfer learning strategy to transfer the pre-trained weights of the EfficientNetB0 model on the Imagenet data set to this model as the initial weights. Model training adopts the Adam optimizer and uses cross-entropy loss. The initial learning rate is 0.001, and exponential decay is used to dynamically adjust the learning rate, with a decay rate of 0.9. The batch size is 32. The number of iteration rounds is 100, and early stopping technology is enabled. Training will stop if the loss does not improve after 10 times.

### 2.4 Evaluation index

In actual production, corn variety classification need to consider accuracy and speed. This study selected accuracy(Acc), precision (P), recall (R), average precision (AP), average Mean average precision (mAP), inference time $I_{t}$ , the formulas are as follows in Equations 8–13:


(8) $$ACC=\frac{TP+TN}{TP+TN+FP+FN}\times 100\%$$

(9) $$P=\frac{TP}{TP+FP}\times 100\%$$

(10) $$R=\frac{TP}{TP+FN}\times 100\%$$

(11) $$AP=\int_{0}^{1}P\cdot \left(R\right)dR\;\;$$

(12) $$mAP=\;\frac{\sum_{i=1}^{n}AP\left(n\right)}{5}\times 100\%$$

(13) $$I_{t}=\frac{t_{N}}{N}$$

Among them, TP represents the number of corn ear images in the test set that were correctly recognized by the model as belonging to the category, and FP represents the number of images of other categories of corn ears that were incorrectly recognized as the current category.TN represents the number of images that are not of the current category and are not recognized by the model as the current category, FN represents the number of images of the current category that are incorrectly identified as ear images of other categories. N is the total number of images, and $t_{N}$ is the total time taken to infer the test set images.

## 3 Results and discussion

### 3.1 Classification and recognition results of corn images by the model

**Figure 6:**
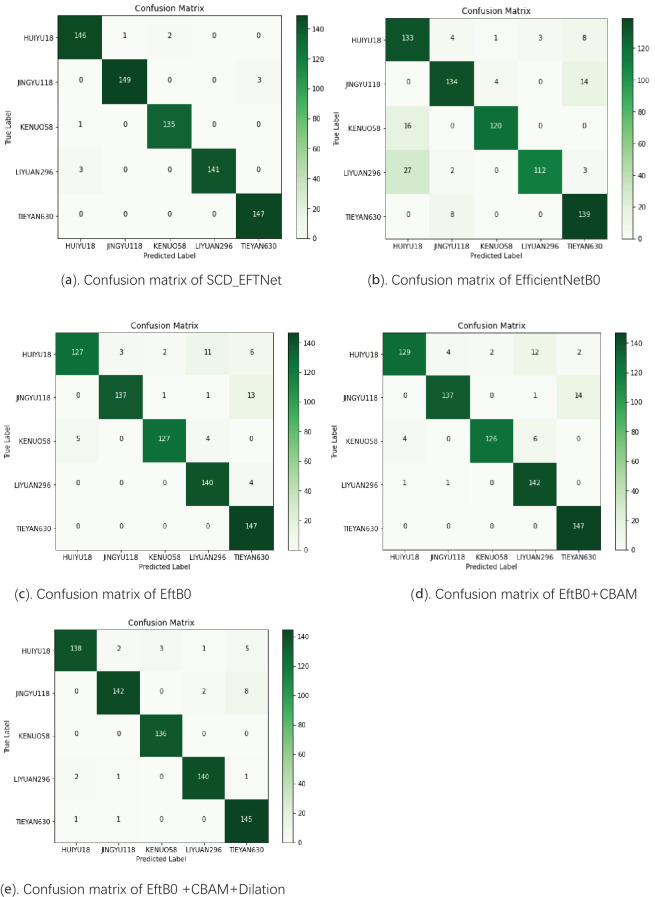
Confusion matrix of different models. The darker the color, the more occurrences of the corresponding predicted-true class combination, and the darker green on the main diagonal indicates that a large number of samples are correctly classified. **(a)** Shows the confusion matrix of the proposed SCD_EFTNet. **(b–e)** Show the confusion matrices after using different strategies in the ablation experiment.

The SCD_EFTNet model was tested on the test set, and the confusion matrix obtained by the model for classifying corn ear images is shown in **Figure 6a**. There are 728 images in the test set, and the model correctly identified 718 images, with a recognition accuracy rate of 98.63%, indicating that the model has good classification and recognition capabilities for corn ear images of different varieties. From a single category perspective, the model also shows good recognition performance. The confusion matrices of other models are shown in the **Figure 6** below. The recognition accuracies of EfficientNetB0, EftB0, EftB0+CBAM, and EftB0+CBAM+Dalition are 87.64%, 93.13%, 93.54%, and 96.29% respectively.

Among the five categories of corn, the model in this article has the best recognition performance on TIEYAN630, with its Precision, Recall and average precision(AP) reaching 98.00%, 100% and 99.97%, respectively. At the same time, the model also performed well in identifying three another corn ears, JINYU118, KENUO58, and LIYUAN296. Judging from the three indicators of P, R, and AP, the above indicators of the three varieties reached 99.33%, 98.03%, 97.37% and 98.54%, 99.26%, 98.59% and 100%, 97.92%, 99.25%, respectively. Although the model is slightly less effective in identifying the ear of variety HUIYU18, the three indicators are all above 95.37%, and the recall rate reaches 97.99%. The results are shown in **Table 2**.

**Table 2:** SCD_EFTNet model evaluation indicators results.

Maize category	Precision P/%	Recall R/%	Average precison AP/%	Inference time I_t/_ms	Mean average precison mAP/%
HUIYU18	97.33	97.99	95.37	0.418	98.11
JINYU118	99.33	98.03	97.37		
KENUO58	98.54	99.26	98.59		
LIYUAN296	100.00	97.92	99.25		
TIEYAN630	98.00	100.00	99.97		

### 3.2 Ablation experiments

In order to verify the effectiveness of the shallow EfficientNetB0 network, adding improved CBAM attention and dilated convolution for corn ear image recognition, this article designed an ablation experiment. The experimental results are shown in **Table 3**. From the results, the average precision mean (mAP) of the shallow model EftB0 after reducing repeated stacking is 8.47% higher than that of EfficientNetB0, the model size is reduced by 9.92MB, the degree of reduction is large, and the inference speed is only about 0.3ms. This is a lightweight model that can process input data and display results faster in practical applications, and is suitable for real-time or delay-sensitive scenarios. After adding CBAM to the shallow model, the mAP increased by 8.9%, the model size was reduced by 9.58MB, and the inference speed was reduced by about 0.1ms (compared to 0.536ms). After further introducing dilation convolution, the mAP increased by 12.89%, and the model size and inference time were the same as after adding CBAM. After continuing to replace the ReLU function in MLP with Swish function, the model size increased by 20.1MB and the inference time almost did not change, but the average accuracy mAP of the model increased by 15.85%.

**Table 3:** Ablation experiment results.

Models	Average precision AP/%					mAP/%	I_t_/ms	Model size/MB
	HUIYU18	JINYU118	KENUO58	LIYUAN296	TIEYAN630			
EfficientNetB0	67.45	80.65	85.48	86.56	91.15	82.26	0.536	16.4
EftB0	82.01	88.20	91.38	94.82	97.22	90.73	0.314	6.48
EftB0+CBAM	83.35	86.96	91.20	96.46	97.83	91.16	0.432	6.82
EftB0+CBAM+Dalition	90.65	91.43	97.87	97.90	97.90	95.15	0.423	6.82
SCD_EFTNet	95.37	97.37	98.59	99.25	99.97	98.11	0.418	36.5

*EftB0 is a simplified model of EfficientNetB0. SCD_EFTNet is the model proposed in this article. It improves CBAM on the basis of EftB0+CBAM+Dalition, that is, it replaces the ReLU function in MLP with the Swish function.

**Figure 7:**
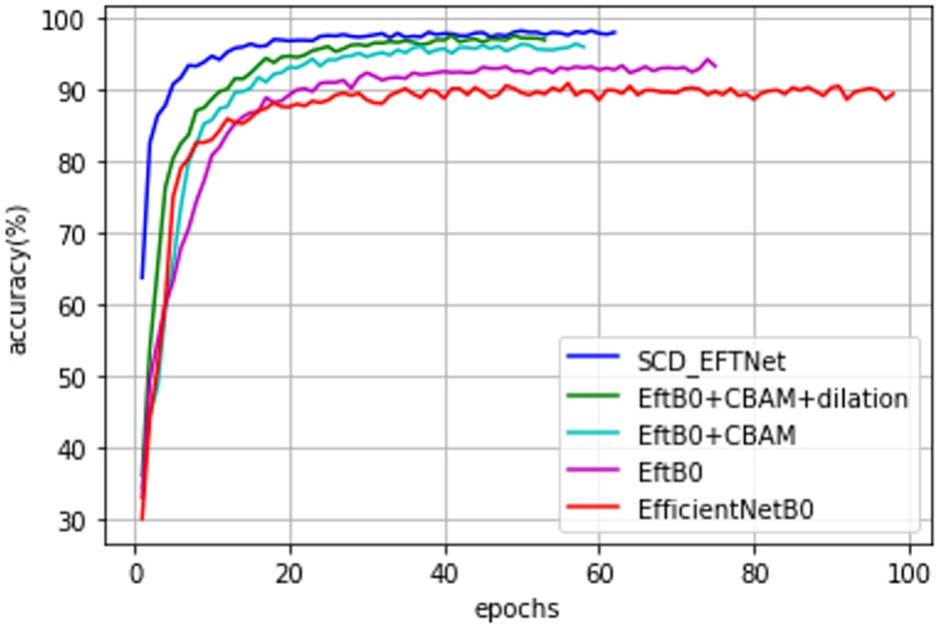
Accuracy curves of different models on training set.

**Figure 8:**
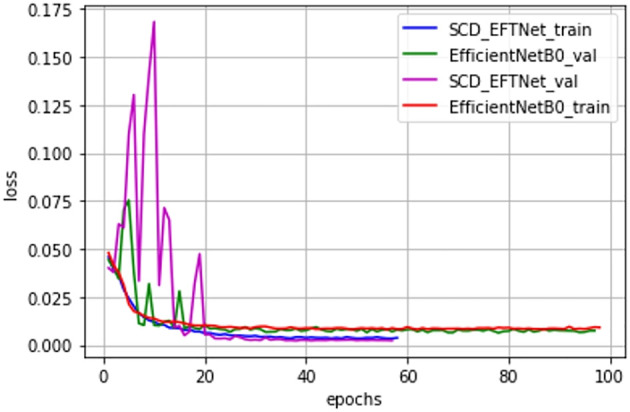
Loss curves of improved model with baseline.

Observe the convergence speed of the improved model during the training process. Compared with the baseline model EfficientNetB0, the streamlined model has been improved after adding attention CBAM and dilated convolution. Especially after improving CBAM, the joint effect is more obvious, the convergence speed of the SCD_EFTNet model is also accelerated, as shown in **Figure 7**. From the perspective of the loss curves in **Figure 8**, the loss value of this model on the training set and verification set is also smaller than before improvement. This is related to the fact that the Swish function can better propagate the gradient during training. We did not perform ablation experiments on Swish alone. The reason for this is that Swish only replaces the original ReLu activation function in the MLP part of the CBAM module, so that it can be consistent with Swish in the MBConv module and give full play to the performance of the Swish function in avoiding gradient vanishing and fast convergence.

### 3.3 Grad-CAM visual analysis

**Figure 9:**
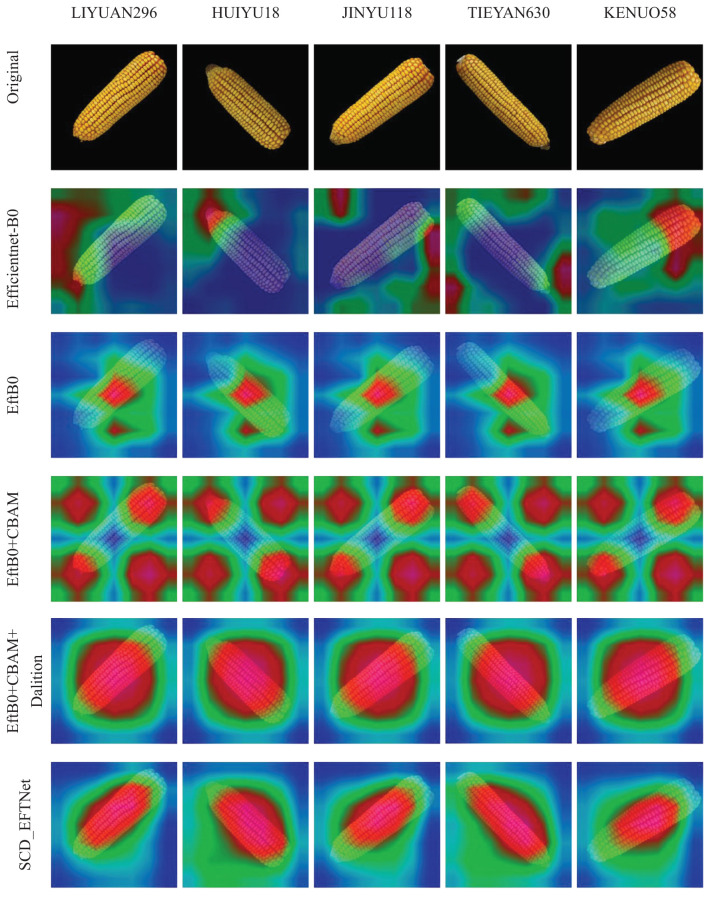
Visualization of the area of interest in corn ear during the model improvement process. In the heat map, red indicates the area of that our model pays more attention to, while blue or green indicates that it pays less attention.

Breeding experts use corn ears to identify different varieties. They generally identify them through phenotypic traits such as outline shape, convex tip size, kernel color, rows per ear, and kernels per row. Different breeds will have different phenotypic characteristics. In this study, Grad-CAM technology (Chattopadhay et al., 2018) was used for visual interpretation and analysis, and visual evaluation of model improvements was performed. The heat map of the improved model is shown in **Figure 9**. It can be seen from the figure that after the initial transfer learning, the model’s areas of interest are messy and scattered, and even focus on some background areas. After the model was simplified, the focus began to shift to the ear, but the area of concern was relatively small. After adding CBAM, the model’s attention range began to increase, mainly at the top and root of the ear, but it was still scattered. After adding dilation convolution, the attention range was concentrated again, but the range was relatively large, which was related to the expansion of its receptive field range. Finally, with the use of the Swish activation function, the model’s focus area is concentrated on the main body of the ear. The heat map shows that the model proposed in this article can accurately extract the characteristic information of the ears.

### 3.4 Comparison with other models

The performance results of different models on the test set are shown in **Table 4**. Overall, in addition to the proposed model, the best performers are MobileNet V2 and DenseNet121, with mAP reaching 93.53% and 93.13% respectively; the worst performance is Inception V3, whose mAP is only 70.11%. The inference time of the proposed model is slightly longer than that of the best-performing MobileNet V2, and the model size is also larger than this model, but its mAP is 4.58% higher. The increase in mAP indicates that the model has enhanced its ability to learn variety-specific features (such as corn cob color distribution and kernel arrangement density). For fine-grained classification tasks such as corn variety identification, it can effectively distinguish varieties with similar morphology. In the variety purity detection scenario, the workload of manual re-inspection can be reduced by 40%.While achieving an overall higher mAP, the proposed model also has a relatively balanced classification performance of a single category of corn varieties, and can better complete the task of classifying and identifying corn varieties.

**Table 4:** Comparison of classification performance of different models on the test set.

Compared models	Average precision AP/%					mAP/%	I_t_/ms	Model size/MB
	HUIYU18	JINYU118	KENUO58	LIYUAN296	TIEYAN630			
VGG16	87.66	86.72	86.80	83.78	84.49	85.89	0.206	537
RestNet34	88.66	89.32	90.98	92.98	91.98	90.78	0.324	85.3
MobileNet V2	94.87	93.94	93.56	94.81	90.45	93.53	0.342	9.19
DenseNet121	80.54	91.49	97.87	97.87	97.87	93.13	0.709	28.5
Inception V3	54.11	57.68	76.30	76.30	86.18	70.11	0.774	101
ShuffleNetV2	45.62	53.30	90.63	90.63	90.63	74.16	0.348	5.23
SCD_EFTNet	95.37	97.37	98.59	99.25	99.97	98.11	0.418	36.5

#### 3.4.1 Bootstrap analysis

Bootstrap methods are particularly valuable in deep learning-based image classification, as they help estimate the uncertainty and variability of performance metrics, especially in cases where data distribution may be complex or imbalanced. We conduct a bootstrap and confidence interval analysis to assess the model’s performance on the evaluation datasets. The test set was sampled with replacement B=1000 times, and the sample size was consistent with the test set size (n=728). The mAP mean was 97.28% and the standard error was 0.32%. Using the quantile method, when the confidence level was 1-α=0.95, the confidence interval of mAP was [96.63%, 99.42%], which was highly consistent with the original test result (98.11%). The Bootstrap estimate and confidence interval of each corn variety are shown in **Table 5**. From the results, it can be seen that they are highly consistent with the results of the SCD_EFTNet model.

**Table 5:** Result of Bootstrap method and confidence interval.

Methods	Average precision AP/%					mAP/%
	HUIYU18	JINYU118	KENUO58	LIYUAN296	TIEYAN630	
SCD_EFTNet	95.37	97.37	98.59	99.25	99.97	98.11
BootStrap	94.23 ± 0.32	96.33 ± 0.42	97.34 ± 0.12	98.23 ± 0.16	98.22 ± 0.23	97.28 ± 0.32
*CI(0.95)	[93.65,94.90]	[95.34,98.24]	[97.45,99.12]	[97.12,99.34]	[97.12,99.45]	[96.63,99.42]

*: CI is confidence interval, the confidence level is 1-α=0.95.

#### 3.4.2 MDS analysis

**Figure 10:**
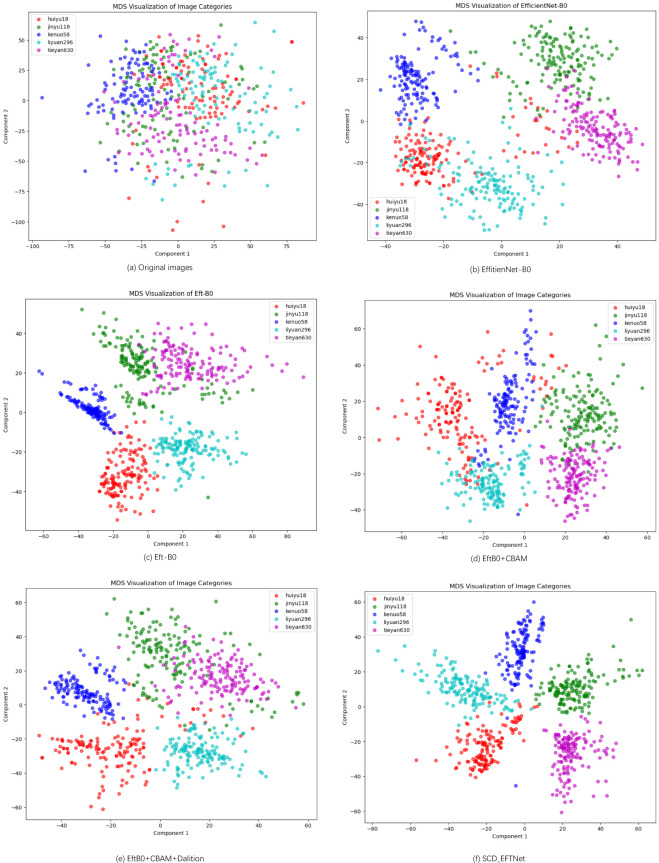
MDS Visualization of five corn varieties. **(a)** is the MDS feature of the original image, and **(b–f)** represent the visualization features of different models in the ablation experiment.

Multidimensional Scaling (MDS) analysis can explore the variation in the dataset across different maize varieties. This can provide visual and quantitative insights into the underlying structure of the data and help identify potential clustering or separation patterns among the varieties. For feature visualization of the original images, we selected the test set images as samples, uniformly adjusted the images to 224*224 pixels and converted them into vectors, used the RestNet50 network to extract deep features, and used the Euclidean distance to calculate the distance matrix of the feature vector. The results after MDS dimensionality reduction are shown in **Figure 10a**.

For other models in our ablation experiment, we extract the deep image features of the penultimate layer of the model, use MDS to reduce the dimension and visualize it. As can be seen from **Figure 10**, the data points are relatively evenly distributed in the two-dimensional space, but there is a certain amount of clustering. The corn ear images of different varieties overlap in some areas, which indicates that these varieties may have similarities in some features. Despite the overlap, most of the data points can still be distinguished by variety. As can be seen from the figures, each part of the model we designed plays a role and better realizes the classification of corn varieties.

### 3.5 Discussion

Traditional varieties identification methods, such as those using molecular isoenzymes and gel electrophoresis of seed storage proteins, have poor reproducibility. The use of molecular marker technology, such as simple sequence repeat (SSR), etc., has high stability and reproducibility for the detection of seed purity and authenticity. However, these methods need to consume expensive materials and design primers, and the experiment preparation and operation procedures are complicated, requiring professional personnel, and the experimental wastes are likely to pollute the environment (Cui et al., 2018; Qiu et al., 2019). Considering the cost and cycle, these methods cannot be adopted by seed processing companies for online detection, thus requiring convenient, fast, and low-cost methods (Xu et al., 2022). The method proposed in this paper can directly identify corn varieties through the RGB images of corn ears. After collecting images of the identified target varieties to train the model, the variety identification can be realized quickly and at low cost.

There are also many efforts to identify corn varieties by using complex professional equipment to obtain kernel phenotypic characteristics, such as near-infrared spectrometers, hyperspectral imagers, scanning electron microscopes, nuclear magnetic resonance, etc. These equipments are generally expensive and complex to operate (Yu et al., 2019). And the obtained data needs to be further corrected, extract effective wavelength spectrum, create database and other operations (Xu et al., 2019; Wang et al., 2021; Zhang et al., 2022). Even if RGB images are used for classification, traditional machine learning classification methods such as SVM, MLP, and KNN (Tu et al., 2022) require complex image preprocessing to extract features. The method proposed in this paper only needs to take pictures with an ordinary digital camera or mobile phone to obtain data. After simple preprocessing, the proposed model can be trained without manual feature extraction, which can simplify the operation steps and achieve good results. The model in this article is lightweight and easy to deploy, and provides a good reference for the development of mobile terminal-based crop germplasm resource identification applications.

Due to the lack of a standard corn ear image database, this paper only collected images of 5 maize varieties. Due to the limited conditions, ear samples were not collected from different planting and promotion areas, under various cultivation conditions and in different years. These conditions may cause weak changes in the phenotypic characteristics of maize ears, which will also interfere with the recognition performance of the model to a certain extent. In the future, we will continue to collect samples under these different conditions to enrich the sample database and enhance the robustness of the model.

Since the images in this article were taken under a uniform black background, they may be different from the actual application scenarios. However, in actual applications, the image acquisition devices of the training set are generally taken in an environment with a relatively controlled background, such as a laboratory, a special light box, a conveyor belt, etc. If the recognition environment is consistent with the training environment, the experimental results will not be much different.

## 4 Conclusion

In order to realize the identification of corn varieties and intelligent screening of ears, this article uses a deep learning model to classify images of five types of corn ears. The following conclusions were drawn through experiments:

1. The improved lightweight EfficientNetB0 model is used to identify corn ear RGB images, which can achieve the same effect as previous efforts in identifying kernel images. Its mAP can reach 98.11%, which can better realize variety identification and intelligent corn ear screening.
2. This article simplifies the EfficientNetB0 model, retaining only an improved MBConv module in each layer, introducing CBAM and replacing the ReLU function in MLP, using dilated convolution. The ablation experiment proves that the above method is effective, and these methods work better together.
3. The proposed model is lightweight. In the corn ear image classification test, the overall performance is superior to mainstream models such as VGG16, MobileNetV2, DenseNet121, RestNet34, etc. This provides a reference for the deployment of mobile terminal applications.

## Data Availability

The datasets presented in this study can be found in online repositories. The names of the repository/repositories and accession number(s) can be found below: [Xu, jinpu (2024), “Corn ear images of five varieties”, Mendeley Data, V1], doi: 10.17632/42z8st838w.1.
